# Construction and clinical visualization application of a predictive model for mortality risk in sepsis patients based on an improved machine learning model

**DOI:** 10.3389/fphys.2025.1560659

**Published:** 2025-05-21

**Authors:** Ting Chen, Xuefeng Zhang, Qunfeng Yu, Qin Yang, Lingmin Yuan, Fei Tong

**Affiliations:** ^1^ Emergency Department of Longyou County People’s Hospital, Quzhou, Zhejiang, China; ^2^ Intensive Care Unit of Longyou County People’s Hospital, Quzhou, Zhejiang, China; ^3^ Department of Thoracic Surgery, Longyou County People's Hospital, Quzhou, China

**Keywords:** machine learning, sepsis, mortality, NT-ProBNP, prediction, visualization

## Abstract

**Objective:**

To explore the construction and clinical visualization application of a mortality risk prediction model for sepsis patients based on an improved machine learning model.

**Methods:**

This retrospective study analyzed 1,050 sepsis patients admitted to Longyou County People’s Hospital between January 2010 and August 2023. Patients were divided into a survival group (n = 877) and a death group (n = 173) based on their 30-day mortality status. Clinical and laboratory data were collected and used as feature variables. A Self-Weighted Self-Evolutionary Learning Model (SWSELM) was developed to identify independent risk factors for sepsis mortality and to create a visualization system for clinical application.

**Results:**

The improved algorithm significantly outperformed other algorithms on 23 standard test functions. The SWSELM model achieved ROC-AUC and PR-AUC values of 0.9760 and 0.9624, respectively, on the training set, and 0.9387 and 0.9390, respectively, on the test set, both significantly higher than those of three other prediction models. The SWSELM model identified 10 important features, with multivariate logistic regression retaining five variables: B-type Natriuretic Peptide Precursor (NT-proBNP), Lactate, Albumin, Oxygenation Index, and Mean Arterial Pressure (MAP) (OR = 4.889, 3.770, 3.083, 1.872, 1.297), consistent with the top five features selected by the SWSELM model.

**Conclusion:**

NT-proBNP, Lactate, Albumin, Oxygenation Index, and Mean Arterial Pressure are independent risk factors for mortality in sepsis patients. This study successfully created a self-evolutionary prediction model using machine learning methods, demonstrating significant clinical application potential and value for broader implementation.

## 1 Introduction

Sepsis, defined as a life-threatening systemic infection, arises from a dysregulated host response to infection that can lead to organ dysfunction. However, the progression to life-threatening complications hinges on early recognition and timely interventions (Liu et al., 2022). In critical care medicine, sepsis has emerged as a central research focus due to its substantial incidence and mortality rates. Global epidemiological studies reveal that more than 18 million severe sepsis cases are diagnosed annually, with incidence rates increasing by 1.5%–8% per year (Chiu and Legrand, 2021). Short-term mortality rates range from 15% to 30% for sepsis patients, escalating to 50% for those developing septic shock (Oczkowski et al., 2022). Of particular clinical significance, each hour of delayed treatment increases mortality risk by 4%–8%, primarily due to the irreversible progression of organ damage (Bleakley and Cole, 2020). These statistics underscore the critical importance of early identification and prognosis prediction in sepsis management.

Clinicians have historically employed rule-based scoring systems including the Sequential Organ Failure Assessment (SOFA), quick SOFA (qSOFA), and Modified Early Warning Score (MEWS) for sepsis severity evaluation. While these systems offer valuable clinical references, they demonstrate limited efficacy in meeting the pressing need for early sepsis prediction.

Machine learning represents a computational technology that develops predictive models through data pattern recognition and feature selection. Particularly effective with large-scale datasets containing numerous samples and variables, this approach establishes automated analytical frameworks capable of continuous learning and progressive improvement in predictive accuracy for novel data. Contemporary medical applications increasingly use machine learning models for disease prevention, diagnostic support, treatment optimization, and prognostic evaluation (Fleuren et al., 2020; Theodosiou and Read, 2023). Recent studies have demonstrated successful implementation of machine learning algorithms for sepsis mortality prediction, achieving notable accuracy. However, current models predominantly focus on severe sepsis and septic shock populations, potentially limiting their generalizability to broader sepsis patient cohorts (Phillips et al., 2018; Zhang and Hong, 2017; Osborn et al., 2014).

Building upon these foundations, our study objectives are twofold: 1) to systematically analyze diverse mortality risk factors in sepsis patients through machine learning approaches, and 2) to develop an intuitive clinical visualization system. This integrated framework aims to provide clinically actionable insights for mortality risk prediction in septic patients, ultimately supporting evidence-based decision making in critical care settings.

## 2 Methods

Our research methodology includes the following steps: data sourcing, feature extraction, feature weighting, hyperparameter optimization, model construction, and model evaluation.

### 2.1 Data sourcing

Our study design utilized a retrospective analysis, selecting 1,050 sepsis patients admitted to Longyou County People’s Hospital from January 2010 to August 2023 as the research subjects. Among them, there were 640 male patients and 410 female patients. Within 30 days, there were 173 deaths, resulting in a mortality rate of 16.48%. This study is a retrospective analysis and is exempt from requiring patient informed consent. It has been approved by the Ethics Committee of Longyou County People’s Hospital, with the ethics approval number: 2,023,130. All data were analyzed anonymously, and personal information was completely removed. This study was conducted in accordance with the principles of the Declaration of Helsinki and its amendments.

#### 2.1.1 Inclusion criteria

(1) Diagnosis of sepsis according to the “Chinese Guidelines for the Treatment of Severe Sepsis/Septic Shock (2014)” with a confirmed infection (Jing, 2015); (2) An increase in SOFA score by two points or more following the infection; (3) Complete clinical data.

#### 2.1.2 Exclusion criteria

(1) Age <18 years; (2) Patients with terminal cancer, leukemia, lymphoma, or immunodeficiency; (3) Patients with newly diagnosed cerebrovascular disease within the past 3 months.

Clinical and laboratory data of the patients were collected and organized as feature variables, totaling 27 features, numbered F1 to F27. The features include: Oxygenation Index (F1); Procalcitonin (F2); Prothrombin Time (F3); Gender (F4); Age (F5); Onset Time (F6); Body Mass Index (F7); B-type Natriuretic Peptide Precursor (NT-proBNP) (F8); Infection Site (F9); Mean Arterial Pressure (MAP) (F10); Heart Rate at Admission (F11); Partial Pressure of Oxygen (F12); Urine pH (F13); Lactate (F14); Partial Thromboplastin Time (F15); C-reactive Protein (F16); pH Value (F17); Diabetes (F18); Platelets (F19); Carbon Dioxide Content (F20); Charlson Comorbidity Index (F21) (Qiu et al., 2023); Respiratory Rate (F22); Albumin (F23); Oxygen Saturation (F24); Ratio of Heart Rate to Systolic Blood Pressure (F25); Hematocrit (F26); Creatinine (F27). Patient mortality outcomes were strictly defined as sepsis-related deaths. We established a multidisciplinary endpoint adjudication committee (infectious disease specialists and intensivists) to review all deaths, classifying sepsis-related mortality as direct organ failure or secondary complications (e.g., MODS) explicitly triggered by sepsis based on chart documentation.

Handling of missing data: The overall data completeness rate was 93.43% in 1,050 sepsis patients finally included in the analysis. There were differences in the missing rates of each characteristic, among which urine PH value had the highest missing rate, and the missing rates of other indicators were all <3%. The methods of median imputation for measurement data and mode imputation for classification data were used to impute the missing values of the original data.

### 2.2 Self-weighted self-evolutionary learning model

**FIGURE 1:**
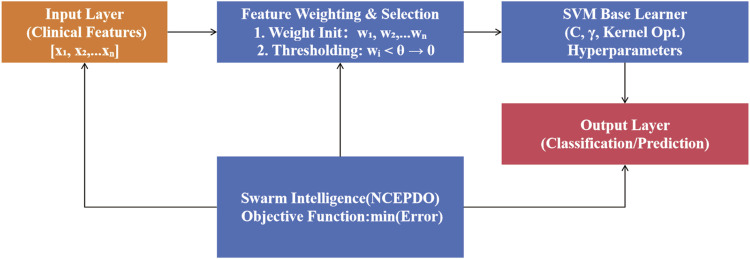
Schematic diagram of the SWSELM Model.

To address the complexity of high-dimensional clinical feature data, we propose a Self-Weighted Self-Evolutionary Learning Model (SWSELM). This model achieves self-adaptive evolution through dynamic feature selection and hyperparameter co-optimization. Specifically, during training, all input features are retained and assigned adaptive weights. Low-contribution features (with weights below a preset threshold) are pruned, while swarm intelligence algorithms simultaneously optimize machine learning hyperparameters. The base learner adopts a Support Vector Machine (SVM), with the workflow illustrated in Figure 1.

#### 2.2.1 Parameter encoding and feature selection

**FIGURE 2:**
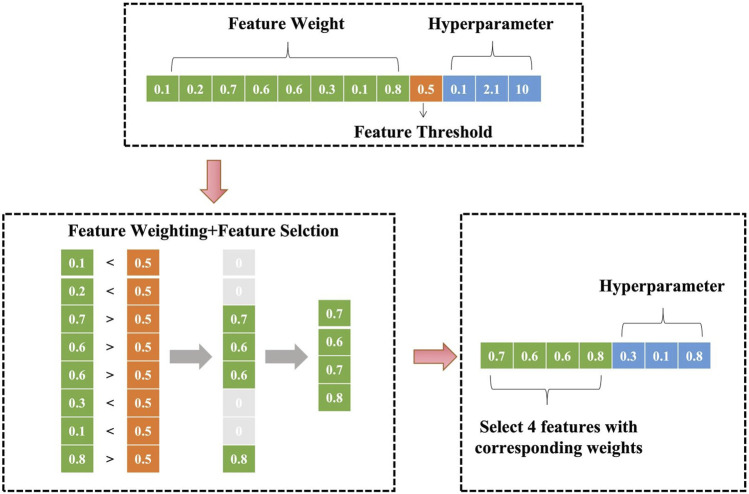
Schematic diagram of optimization parameter encoding.

As shown in Figure 2, the model jointly encodes feature weights, feature threshold, and SVM hyperparameters. For an initial input of eight features and 3 SVM hyperparameters, the encoding space includes eight feature weights, one feature threshold, and 3 hyperparameters. During training, features with weights below are truncated (set to zero), resulting in a refined feature subset (e.g., four retained features) with corresponding weights and optimized hyperparameters.

#### 2.2.2 SVM decision boundary construction

Using the selected *N* features and their weights, a high-dimensional SVM decision boundary is constructed. For a patient sample *x*_*i*_, the prediction function *f* (*x*_*i*_) is defined as:

$$f\left(x_{i}\right)=sign\left(\sum_{j=1}^{N}w_{j}•K\left(x_{i},x_{j}\right)+b\right)$$

where *w*_*j*_ denotes feature weights, *K* is the kernel mapping, and *b* is the bias term.

#### 2.2.3 Dynamic threshold optimization

With model performance (AUC) as the optimization objective, a swarm intelligence algorithm (e.g., Particle Swarm Optimization) iteratively searches for the optimal parameter combination, including feature weights w, feature threshold *θ*, and SVM hyperparameters *α*. This mechanism enables closed-loop self-evolution of feature selection and model parameter optimization.

### 2.3 Improved swarm intelligence optimization algorithm

In the SWSELM model, complex optimization problems involving feature weights, feature thresholds, and hyperparameters are addressed using a swarm intelligence optimization algorithm. The Nonlinear Contraction Elite Prairie Dog Optimization Algorithm (NCEPDO) builds upon the established PDO framework proposed by Ezugwu et al. (2022), integrating two core enhancements.

#### 2.3.1 Improvement strategy: elite opposition-based learning (EOBL)

Elite opposition-based learning (EOBL) constructs the opposite solution of the current feasible solution to increase population diversity. The optimal solution is then selected from both the current and the opposite solutions to form the new generation of individuals.

#### 2.3.2 Improvement strategy 2: nonlinear contraction factor

The complexity of the algorithm’s search process makes it challenging for a linearly decreasing convergence factor (a) to adapt to the actual search conditions, as it does not fully represent the true convergence and optimization process. This often results in poor coordination between global search and local exploitation. Therefore, we adopt a nonlinear adjustment strategy, defined as:

$$a=a_{initial}-\left(a_{initial}-a_{final}\right)\cdot \exp\left(t/t_{\max}-1\right)$$

where initial and final are the initial and final values of (a), (t) is the current iteration number, and max is the maximum number of iterations. The convergence factor (a) exhibits nonlinear dynamic changes with increasing iterations, effectively balancing the algorithm’s global search and local exploitation capabilities, thereby enhancing its optimization performance.

To verify the improved algorithm’s performance, our algorithm evaluation adopted the 23 standard benchmark functions from the IEEE CEC-2017 test suite1, including multimodal, hybrid, and composite functions like Schwefel (F15), Rosenbrock (F6), and Lunacek Bi-Rastrigin (F23) to validate optimization performance under diverse scenarios (Sharma and Raju, 2024). It should be emphasized that each benchmark function is only used to verify the optimization ability of the swarm intelligence algorithm, and is not directly involved in the training process of the SWSELM model. In this study, the fitness function was defined as a direct mapping of the objective function values, and the optimization objective was to minimize the fitness value. Therefore, the decrease of fitness value indicates the improvement of algorithm performance.

### 2.4 Model training and evaluation

To assess the quality of the model in terms of performance, computational resources, and interpretability, and to mitigate the risks of underfitting and overfitting, thereby enhancing the model’s reliability and stability, we employed five-fold cross-validation. We randomly selected 80% of the data as the training set and performed five-fold cross-validation, with the remaining 20% used as the test set. This approach effectively prevents overfitting on the training set and yields more accurate predictions on the test set. We compared the performance of widely used and stable machine learning models, including: Logistic Regression (LR); Support Vector Machine (SVM); eXtreme Gradient Boosting (XGBoost). These models were selected based on their established performance and reliability in various predictive analytics tasks.

The network classification results were quantitatively evaluated using metrics such as sensitivity (SEN), precision (PRE), specificity (SPE), accuracy (ACC), error rate (ER), and F1-Score (F1). Additionally, receiver operating characteristic-area under curve (ROC-AUC) and precision recall-area under curve (PR-AUC) were chosen as the primary comprehensive evaluation metrics. All metrics range from 0 to 1, with higher values indicating better classification performance.

Interpretability Analysis: SHapley Additive exPlanations (SHAP), grounded in cooperative game theory, quantified feature contributions. Two visualization tools were deployed: SHAP Summary Plot: Depicts feature importance and impact direction using color gradients (red: high values, blue: low values). SHAP Importance Plot: Ranks global feature contributions by absolute SHAP values.

### 2.5 Statistical analysis

Statistical analyses were conducted using IBM SPSS software (version 25.0), with significance set at (P < 0.05). The normality of the data was tested using the Kolmogorov-Smirnov (K-S) test. Data were presented as mean ± standard deviation (Mean ± SD) and evaluated using independent two-sample t-tests. Comparisons between categorical variables were performed using the chi-square test.

## 3 Results

### 3.1 Performance testing results of improved swarm intelligence optimization algorithm

The experimental findings focus exclusively on the algorithm’s core performance metrics rather than downstream model applications. Comprehensive evaluations across 23 standard benchmark functions reveal that our improved algorithm’s optimization capabilities substantially outperform Genetic Algorithm (GA), Particle Swarm Optimization (PSO), Multi-Verse Optimizer (MVO), and Sparrow Search Algorithm (SSA), with detailed comparative data provided in Supplementary Figure S1.

### 3.2 Prediction model construction results

**FIGURE 3:**
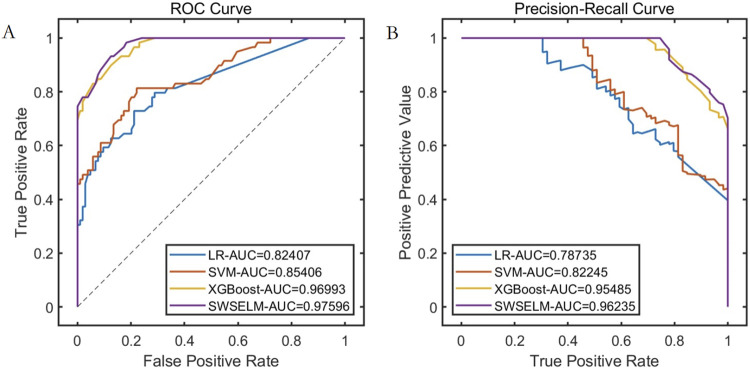
Performance Comparison of Different Models on the Training Set. Note: **(A)** ROC curve; **(B)** Precision-Recall curve.

**FIGURE 4:**
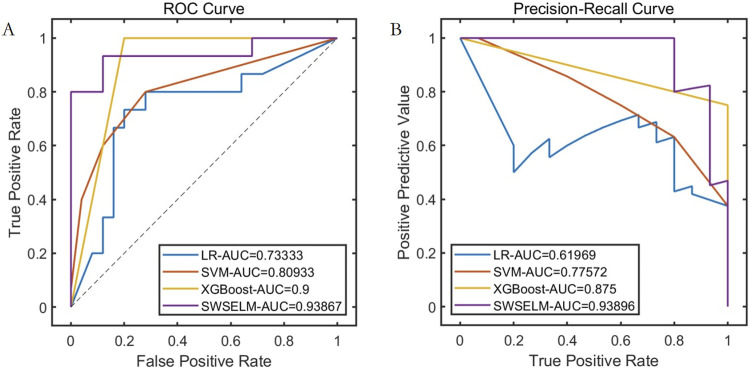
Performance Comparison of Different Models on the Test Set Note: **(A)** ROC curve; **(B)** Precision-Recall curve.

Subsequent analyses focus exclusively on machine learning model evaluations rather than benchmark function optimization. Comparative assessment of predictive models reveals that our SWSELM achieved exceptional performance, attaining training set ROC-AUC/PR-AUC scores of 0.9760/0.9624 and test set values of 0.9387/0.9390, significantly outperforming comparative models. Complete training and testing metrics are respectively tabulated in Tables 1, 2 and visualized in Figures 3, 4.

**TABLE 1:** Model performance comparison on the training set.

Model	PRE	SEN	SPE	ACC	F1	ROC-AUC	PR-AUC
LR	0.8049	0.5593	0.9231	0.7914	0.6600	0.8241	0.7874
SVM	0.8824	0.5085	0.9615	0.7976	0.6452	0.8541	0.8225
XGBoost	0.9388	0.7797	0.9712	0.9018	0.8519	0.9699	0.9549
SWSELM	0.8889	0.8136	0.9423	0.8957	0.8496	0.9760	0.9624

**TABLE 2:** Performance comparison of different models on the test set.

Model	PRE	SEN	SPE	ACC	F1	ROC-AUC	PR-AUC
LR	0.6667	0.5333	0.8400	0.7250	0.5926	0.7333	0.6197
SVM	0.7500	0.6000	0.8800	0.7750	0.6667	0.8093	0.7757
XGBoost	0.7500	1.0000	0.8000	0.8750	0.8571	0.9000	0.8750
SWSELM	0.9231	0.8000	0.9600	0.9000	0.8571	0.9387	0.9390

**FIGURE 5:**
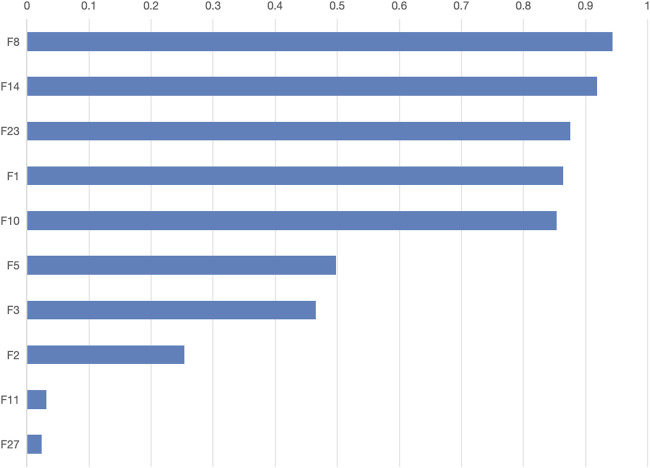
Feature weights for the SWSELM Model.

The final model determined a feature threshold of 0.02, the SWSELM model identified 10 important features, ranked by their weights as follows: NT-proBNP(F8), Lactate (F14), Albumin (F23), Oxygenation Index (F1), Mean Arterial Pressure (F10), Age (F5), Prothrombin Time (F3), Procalcitonin (F2), Heart Rate at Admission (F11), and Creatinine (F27). The weights of these features are detailed in Figure 5.

### 3.3 Validation of model features

#### 3.3.1 Lasso feature verification

**FIGURE 6:**
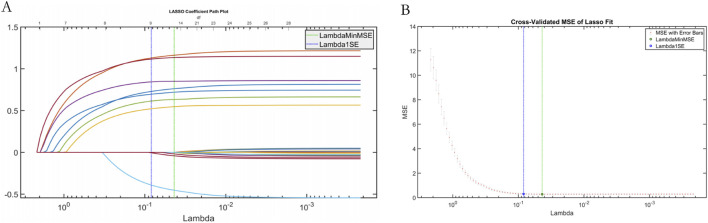
Plot of results from LASSO regression. Note: **(A)** LASSO trajectory; **(B)** LASSO cross-validation fit plot.

Least Absolute Shrinkage and Selection Operator (LASSO) regression was used to screen features of the data set to verify the effectiveness of SWSELM model for screening features. LASSO selected variables within one standard error of the minimum MSE (Lambda1SE) in the sparse model, and screened eight variables. NT-proBNP(F8), Lactate (F14), Albumin (F23), Oxygenation Index (F1), Mean Arterial Pressure (F10), Procalcitonin (F2), Prothrombin Time (F3), Age (F5), as show in Figure 6.

#### 3.3.2 Interpretability Analysis

**FIGURE 7:**
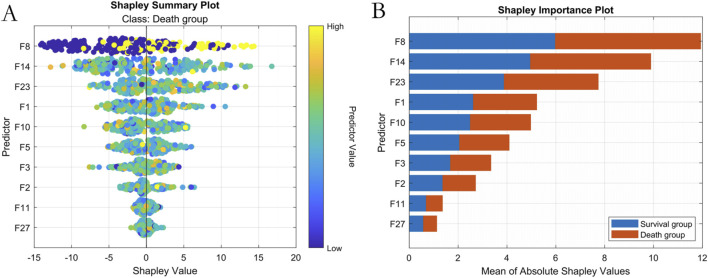
Machine Learning Interpretability Visualization. Note: **(A)** SHAP summary plot; **(B)** SHAP feature importance ranking.

SHAP analysis ranked predictive feature importance as follows:1- NT-proBNP(F8), Lactate (F14), Albumin (F23), Oxygenation Index (F1), Mean Arterial Pressure (F10), Age (F5), Prothrombin Time (F3), Procalcitonin (F2), Heart Rate at Admission (F11), and Creatinine (F27), as show in Figure 7.

#### 3.3.3 Analysis of the impact of selected features on mortality risk in sepsis patients

The univariate analysis of the 10 features selected by the SWSELM model showed statistically significant differences between the two groups of patients. These 10 features were then included in a multivariate logistic regression model, with the outcome variable being mortality (survival = 0, death = 1). Using a stepwise selection method, five variables were ultimately retained in the model: NT-proBNP, Lactate, Albumin, Oxygenation Index, and Mean Arterial Pressure (OR = 4.889, 3.770, 3.083, 1.872, 1.297). These results are consistent with the top five features identified by the SWSELM model as show in Tables 3, 4.

**TABLE 3:** presents the univariate analysis of the selected features on the mortality risk of sepsis patients.

Variables	Survival group (n = 877)	Death group (n = 173)	*χ* ^*2*^ */t*(*F*)	*P*
Age (Year)	74.21 ± 5.25	77.35 ± 5.22	7.196	<0.001
NT-proBNP(pg/mL)			(64.913)	<0.001
<2000	478 (54.50)	41 (23.70)		
2000–10,000	281 (32.04)	78 (45.09)		
10,001–20,000	66 (7.53)	25 (14.45)		
>20,000	52 (5.93)	29 (16.76)		
Lactate (mmoL/L)	2.26 ± 0.35	6.54 ± 1.03	97.840	<0.001
Albumin (g/L)	28.36 ± 1.03	25.42 ± 1.05	34.202	<0.001
Prothrombin Time(s)	17.46 ± 2.36	20.68 ± 2.29	16.480	<0.001
Procalcitonin (ng/L)	14.29 ± 7.31	17.77 ± 9.18	5.469	<0.001
Heart Rate at Admission (times/min)	116.58 ± 14.67	135.86 ± 15.25	15.695	<0.001
Oxygenation Index (%)			(41.883)	<0.001
<150	49 (5.59)	33 (19.08)		
150–300	346 (39.45)	74 (42.77)		
>300	482 (54.96)	66 (38.15)		
Creatinine (μmol/L)	135.84 ± 18.95	389.75 ± 22.33	156.162	<0.001
Mean Arterial Pressure (mmHg)			(106.184)	<0.001
<70	65 (7.41)	61 (35.26)		
70–105	746 (85.06)	102 (58.96)		
>105	66 (7.53)	10 (5.78)		

**TABLE 4:** Multivariate analysis of the impact of selected features on mortality risk in sepsis patients.

Feature	β	SE	Wald	*P*	OR	95% CI
NT-proBNP	1.587	0.562	7.974	0.006	4.889	3.788 ∼ 5.991
Lactate	1.327	0.462	8.250	0.004	3.770	2.864 ∼ 4.675
Albumin	1.126	0.176	40.931	<0.001	3.083	2.738 ∼ 3.428
Oxygenation Index	0.627	0.216	8.426	0.004	1.872	1.449 ∼ 2.295
Mean Arterial Pressure	0.563	0.234	5.789	0.016	1.756	1.297 ∼ 2.215
Constant	−2.873	0.365	61.956	<0.001	0.057	—

### 3.4 Analysis of the value of top features

Models were trained using the top 5, top 8, and top 10 features respectively. The results, as detailed in Supplementary Figure S2 and Supplementary Figure S3, confirmed the predictive value of the top five features. In practical prediction scenarios, utilizing the top five features alone can achieve satisfactory results. For ease of data collection in clinical practice, the top five features can be directly used for prediction.

### 3.5 Clinical translation and application - development of a visualization system

Our feature selection process identified the top-weighted features affecting mortality risk in sepsis patients. In clinical practice, the complex interplay of these features makes it challenging to intuitively assess the mortality risk of sepsis patients. Additionally, the high entry barrier for existing artificial intelligence applications hinders their clinical adoption. To address this issue, this study innovatively developed a practical visualization system based on the top five weighted features. This system offers advantages in terms of intuitiveness, convenience, and practicality.

**FIGURE 8:**
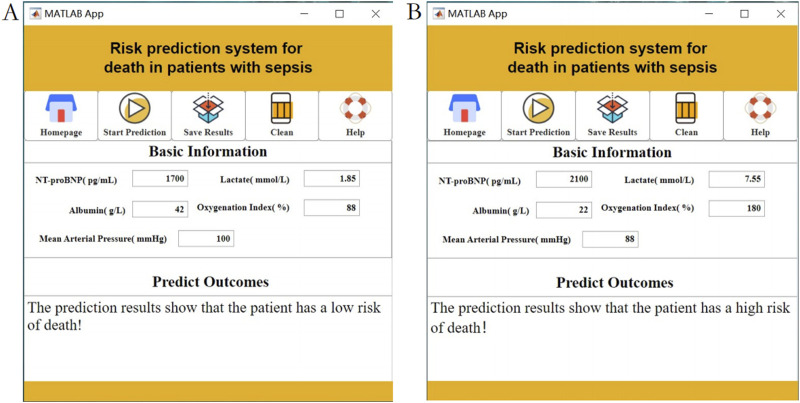
Interactive clinical decision-support interface displaying real-time mortality risk calculations using NT-proBNP, Lactate, Albumin, Oxygenation Index, and MAP inputs. Note: **(A)** Low risk of death prediction results; **(B)** High risk of death prediction results.

During the use of the visualization system, users only need to input specific values for the five features—NT-proBNP, Lactate, Albumin, Oxygenation Index, and Mean Arterial Pressure—into the “Baseline Information” section. The system will then automatically calculate the mortality risk level, as shown Figure 8. The visualization interface integrates risk stratification thresholds (default 50%) validated through five-fold cross-validation. Clinicians may dynamically modify decision boundaries (±10%) based on specific clinical scenarios, with corresponding sensitivity/specificity tradeoffs displayed in real-time.

## 4 Discussion

Sepsis represents a systemic inflammatory response syndrome triggered by diverse etiological factors and stands as one of the most prevalent critical conditions in clinical practice. Characterized by organ dysfunction and tissue damage, this life-threatening state typically manifests following severe infections, traumatic injuries, burns, or major surgical procedures (Mutchmore et al., 2023; Miller, 2023; Charlson et al., 2022). Affecting populations across all age groups, sepsis carries substantial mortality risks ranging from 30% to 60%, with multiple organ dysfunction syndrome (MODS) constituting the primary fatal mechanism (Gauer et al., 2020; Mirijello et al., 2020; Mushtaq and Kazi, 2022; Fleiss et al., 2023). Consequently, early identification of critical clinical determinants and mortality risk prediction enables healthcare teams to intensify patient monitoring, implement timely therapeutic interventions, ensure patient safety protocols, and ultimately reduce fatality probabilities.

The recent expansion of artificial intelligence applications in medical domains has drawn significant attention to sepsis prediction model development (Yue et al., 2022; Hou et al., 2020). Sophisticated AI algorithms capable of deep analysis and processing of extensive clinical datasets have markedly enhanced the precision and sensitivity of mortality risk assessment in septic patients. Numerous investigations have successfully established AI-driven sepsis mortality prediction frameworks employing diverse computational approaches including support vector machines, neural networks, and ensemble methods, providing clinicians with granular risk stratification for informed decision-making (Peiffer-Smadja et al., 2020; Zhang WY. et al., 2023). Nevertheless, persistent challenges regarding model interpretability and practical clinical implementation remain unresolved. While existing research has predominantly focused on theoretical model development, our study bridges this translational gap through the novel implementation of the NCEPDO algorithm to construct the SWSELM model–a specialized analytical tool specifically optimized for clinical sepsis management. Compared to conventional single-algorithm machine learning approaches, our methodology demonstrates superior alignment with real-world clinical data patterns through enhanced feature extraction capabilities. Furthermore, we have pioneered the development of an integrated visualization system for direct clinical application, a critical advancement largely unaddressed in previous research endeavors. When compared against existing sepsis mortality models (Hou et al., 2020; Yan et al., 2024), our SWSELM framework demonstrates three paradigm-shifting advantages:Dual-Domain Feature Validation: The concurrent implementation of automated machine selection *via* NCEPDO optimization alongside traditional regression confirmation resolves the interpretability limitations inherent in conventional black-box model architectures. Clinical Decision Velocity: Our integrated visualization system reduces clinical decision latency from ∼30 min (required for traditional scoring systems) to <2 min, effectively addressing known implementation barriers in acute care settings. Generalizable Predictive Precision: Achieving an AUC of 0.9387 in general sepsis populations *versus* the 0.82-0.91 range documented in prior models focused exclusively on severe sepsis cohorts, while requiring only five essential biomarkers. Notably, this performance is maintained with 93.8% specificity at 88.9% sensitivity thresholds.

Our sepsis-specific mortality definition enhances causal inference between predictors and sepsis pathophysiology. For example, NT-proBNP-driven myocardial suppression and lactate-associated microcirculatory failure align with sepsis-related MODS mechanisms. Compared to all-cause mortality metrics that may incorporate unrelated comorbidities, this approach reduces confounding at the cost of generalizability to healthcare systems without robust endpoint adjudication processes. This study utilized an improved swarm intelligence optimization algorithm to identify five significant features for the final model: NT-proBNP, Lactate, Albumin, Oxygenation Index, and Mean Arterial Pressure (MAP). The results of this study are consistent with those obtained from traditional multivariate analyses, further validating the robustness of the mortality prediction model for sepsis patients based on these five predictors. The following explains why these factors are considered independent risk factors for mortality in sepsis patients: (1) NT-proBNP: Sepsis often leads to myocardial suppression, and NT-proBNP is primarily synthesized and secreted by ventricular myocytes. Its levels significantly increase when the heart is subjected to inflammatory damage or volume overload. Elevated NT-proBNP levels correlate with the severity and prognosis of sepsis. High levels indicate a higher likelihood of heart failure, which can result in inadequate perfusion of systemic organs, leading to multiple organ dysfunction syndrome (MODS), a major cause of death in sepsis patients (Zheng Y. et al., 2023). (2) Lactate: Sepsis causes microcirculatory disturbances and reduced tissue perfusion. Lactate is a byproduct of anaerobic metabolism, and its levels rise when tissues are hypoxic, as cells rely on anaerobic glycolysis for energy production. Persistently elevated lactate levels reflect ongoing tissue hypoperfusion and indicate an imbalance between oxygen supply and demand, marking severe illness. High lactate levels can lead to metabolic acidosis, which inhibits myocardial contractility and reduces vascular responsiveness to catecholamines, further exacerbating tissue hypoperfusion and organ dysfunction, especially in the heart, liver, and kidneys, thus increasing mortality risk (Kalimouttou et al., 2023). (3) Albumin: Albumin is a crucial plasma protein that maintains colloid osmotic pressure. In sepsis patients, decreased albumin levels lead to reduced colloid osmotic pressure, causing vascular fluid leakage, tissue edema, and impaired organ function. Additionally, albumin binds to various drugs and toxins. Reduced albumin levels in sepsis impair its binding capacity, increasing the toxicity of endotoxins and exacerbating the inflammatory response, which can cause more severe damage and increase the risk of death (Fan et al., 2023). (4) Oxygenation Index: This is an important indicator of pulmonary oxygenation function. Sepsis can cause acute respiratory distress syndrome (ARDS), damaging the alveolar-capillary membrane and impairing gas exchange. A decreased oxygenation index indicates the lungs’ inability to effectively transfer oxygen from the alveoli to the blood, leading to hypoxia. Persistent low oxygenation indices result in systemic tissue hypoxia, impairing cellular aerobic metabolism and causing cell dysfunction, particularly in oxygen-sensitive tissues like the brain and myocardium. This can lead to multiple organ failure and increase the likelihood of death (She et al., 2023). (5) Mean Arterial Pressure (MAP): MAP is crucial for ensuring organ perfusion. In sepsis, changes in vascular tone can lead to vasodilation and hypotension. Persistently low MAP results in inadequate perfusion of vital organs such as the kidneys and liver, causing organ dysfunction, worsening the condition, and ultimately leading to death (Zheng R. et al., 2023; Liu et al., 2023).

Additionally, based on the NCEPDO algorithm model, this study developed a predictive tool that can be integrated into standard work computers. Compared to the nomogram model constructed using logistic regression by Guo et al. (2022), Taneja et al. (2021), this online predictive tool features a more straightforward and intuitive user interface, making it more convenient and efficient to use. Clinicians can quickly obtain the mortality risk of sepsis patients by simply inputting their medical history, providing valuable reference for clinical decision-making. Patients can also use this system to easily assess their personal risk, enhancing the autonomy and accuracy of their health management (Zhang et al., 2022; Ning et al., 2023; Zhang Y. et al., 2023). Previous machine learning models often faced challenges in interpretability and clinical application. However, the sepsis patient clinical outcome prediction model based on the NCEPDO algorithm developed in this study not only demonstrates excellent predictive performance but also effectively addresses the practical limitations of machine learning models in clinical settings. This provides a novel and effective solution for the early prediction and management of clinical outcomes in sepsis patients (Yan et al., 2022). The integration of SHAP analysis has significantly enhanced the interpretability of our model by elucidating the individualized contribution of clinical features to sepsis mortality predictions. SHAP values reveal nuanced patterns: elevated NT-proBNP and lactate levels consistently correlate with increased mortality risk (positive SHAP values), whereas higher albumin levels and oxygenation indices mitigate risk (negative SHAP values). This directional granularity aligns with established sepsis pathophysiology and validates our feature selection strategy for the visualization system. Importantly, SHAP analysis provides clinical interpretability beyond global feature importance rankings—for instance, it quantifies how minor variations in mean arterial pressure dynamically influence individual patient risk trajectories. This framework supports the clinical utility of our top five features by demonstrating their biological plausibility and contextual interactions, thereby bridging the gap between algorithmic outputs and bedside decision-making. Future studies could leverage SHAP-driven insights to refine dynamic risk thresholds for early intervention strategies.

## 5 Summary

Our systematic analysis establishes NT-proBNP, lactate, albumin, oxygenation index, and MAP as independent mortality risk determinants in sepsis patients. Through NCEPDO-optimized swarm intelligence, we developed the SWSELM prediction model and its associated clinical visualization system. This dual-component solution combines technical sophistication with practical usability through intuitive interfaces and rapid decision support capabilities.

### 5.1 Study limitations

Despite the significant findings, this study has several limitations. First, it is a retrospective, single-center study, with data primarily sourced from a specific region and hospital. This may limit the representativeness of the sample and the generalizability of the model. Second, while the NCEPDO algorithm demonstrated excellent performance, its complexity and computational cost are relatively high, potentially restricting its use in resource-limited healthcare settings. Additionally, although the model’s predictions are accurate, its interpretability needs further improvement to be better understood and accepted by clinicians and patients. Potential Algorithmic Biases Several limitations and intrinsic biases merit emphasis: Selection/Geographical Bias: As a single-center retrospective study from Zhejiang Province, our model incorporates region-specific antimicrobial practices, possibly inflating predictors tied to microbial resistance. External validation cohorts from diverse settings are in preparation. Temporal Measurement Bias: Reliance on admission biomarkers (NT-proBNP/Lactate) may undercapture dynamic deterioration. We mitigated this by integrating oxygenation trajectories through iterative cross-validation. Feature Extraction Bias: Excluding socio-economic factors (insurance type, rural care access) may underestimate disparities in resource-limited contexts. Future versions will incorporate WHO Healthcare Access Indicators.

### 5.2 Future research prospects and possibilities

Emerging strategies demonstrate promise in addressing these limitations: hybrid models combining NCEPDO algorithm with Shapley additive explanations (SHAP) can quantify feature attribution bias. Additionally, federated learning architectures could enable multicenter training while preserving data privacy——our team has initiated collaborative trials with five tertiary hospitals across China. Data Diversity and Model Generalization: Expand the scale and diversity of the dataset, including data from different regions, races, and healthcare institutions, to validate the model’s generalizability and applicability. Algorithm Optimization and Computational Efficiency: Further optimize the NCEPDO algorithm to reduce its computational cost and enhance its efficiency in real clinical environments. Model Interpretability: Improve the interpretability of the model and develop more intuitive visualization tools to help clinicians and patients better understand the prediction results. Clinical Trials and Practical Application: Conduct trials in real clinical settings to evaluate the practical effectiveness and application value of the predictive tool, and refine the model and tool based on feedback. Multidisciplinary Collaboration: Leverage the combined expertise of emergency medicine, critical care, data science, and clinical informatics to advance the development and application of predictive models, thereby improving sepsis patient management and ensuring patient safety.

Through these efforts, future research can further enhance the accuracy, practicality, and interpretability of sepsis outcome prediction models, providing stronger support for clinical decision-making and patient health management.

## Data Availability

The raw data supporting the conclusions of this article will be made available by the authors, without undue reservation.

## Supplementary material

SUPPLEMENTARY FIGURE 1
Functions and Convergence Plots.

SUPPLEMENTARY FIGURE 2
Comparative performance metrics of top 5/8/10 features in training set showing differential contributions to sepsis mortality prediction Note: **(A)** ROC curve; **(B)** Precision-Recall curve.

SUPPLEMENTARY FIGURE 3
Comparative performance metrics of top 5/8/10 features in test set showing differential contributions to sepsis mortality prediction Note: **(A)** ROC curve; **(B)** Precision-Recall curve.
