# Supplementary figures and images for: Construction and clinical visualization application of a predictive model for mortality risk in sepsis patients based on an improved machine learning model
Source: Front Physiol. 2025 May 21;16:1560659. doi: 10.3389/fphys.2025.1560659 (PMC12133877; doi:10.3389/fphys.2025.1560659)

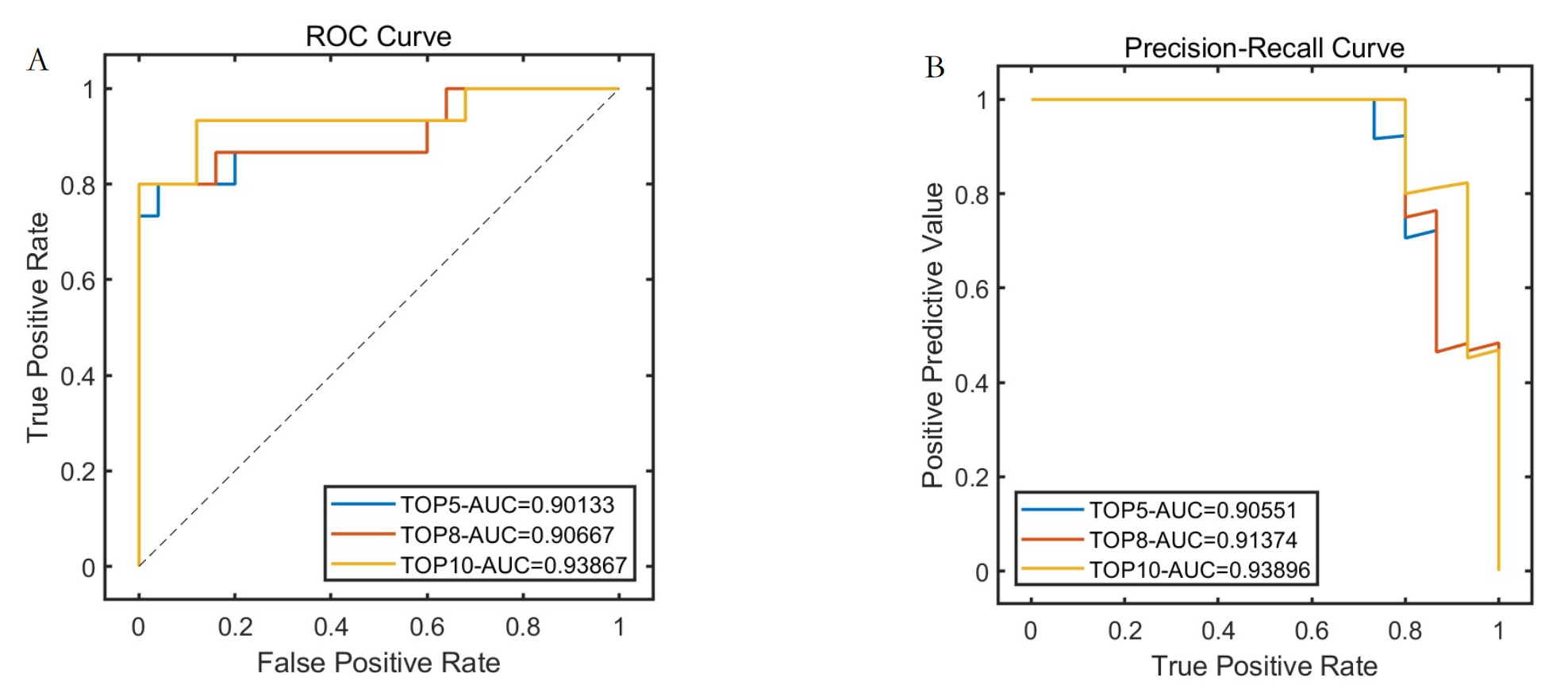

Supplement: Supplementary file 1 [file Image3.jpeg]

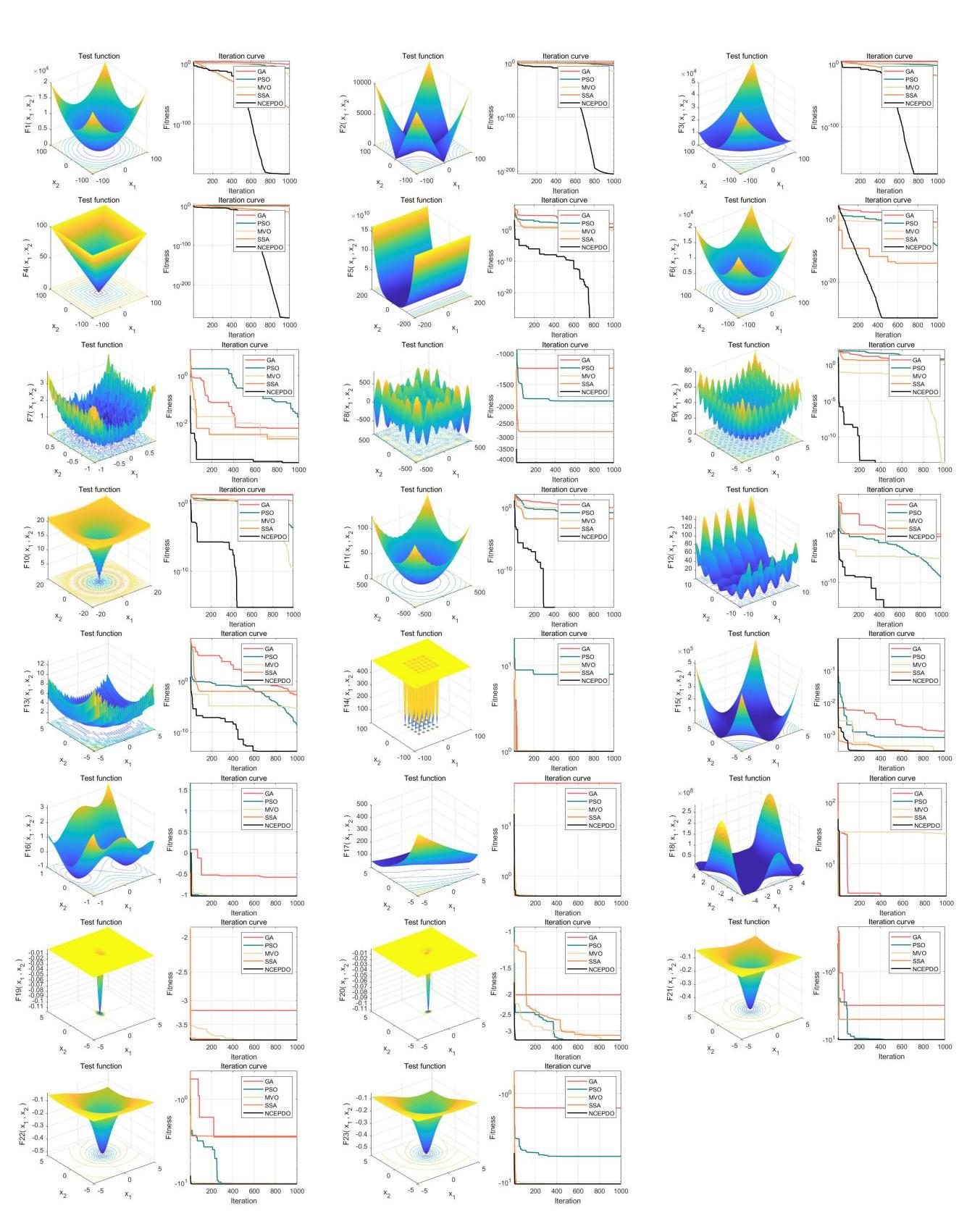

Supplement: Supplementary file 2 [file Image1.jpeg]

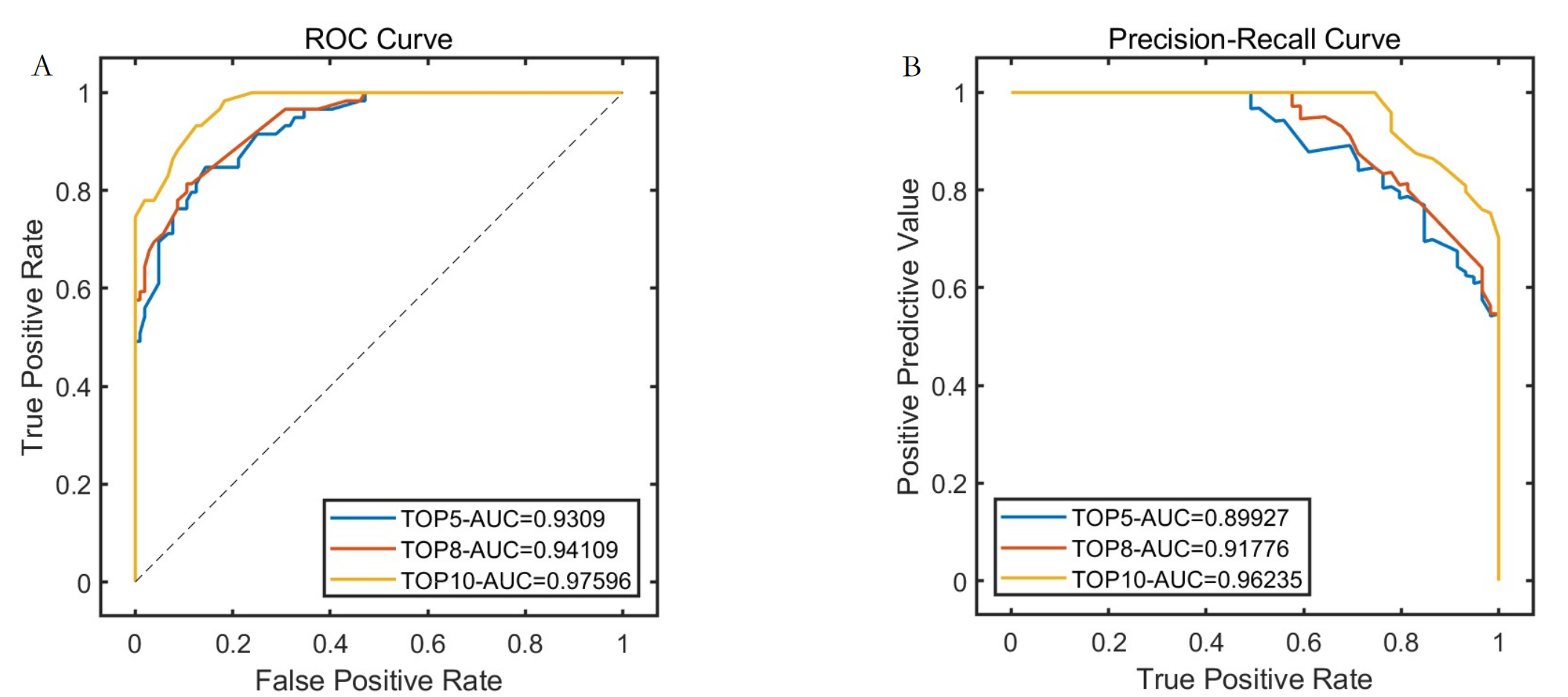

Supplement: Supplementary file 3 [file Image2.jpeg]
